# Chautauqua County Notes

**Published:** 1897-06

**Authors:** 


### CHAUTAUQUA COUNTY NOTES.

DRS. WILLIAM M. BEMUS and Laban Hazeltine, of Jamestown, and Dr. S. P. McCray, of Clymer, constitute the new board of pension examining surgeons for Chautauqua county, with headquarters at Jamestown.

---

DR. G. E. ELLIS, of Brocton, has been compelled to take a rest from his professional work on account of an attack of glycosuria. He has improved during the past month and expects soon to be able to resume his practice.

---

DR. W. A. PUTNAM, of Westfield, has moved out to his farm, near Silver Creek, to recuperate his health following an attack of pneumonia, from which he suffered in November, 1896.

The County Medical Society is arranging for an evening session at its annual meeting in July, at which time a paper will be read on the subject and exhibition of the X-rays given.

---

Dr. S. C. Green, class of '95, University of Buffalo, has located at Panama, N. Y. The profession in Chautauqua county gladly welcomes him into his chosen field of labor.

